# Axonal Transport Defects in a Mitofusin 2 Loss of Function Model of Charcot-Marie-Tooth Disease in Zebrafish

**DOI:** 10.1371/journal.pone.0067276

**Published:** 2013-06-26

**Authors:** Anna L. Chapman, Ellen J. Bennett, Tennore M. Ramesh, Kurt J. De Vos, Andrew J. Grierson

**Affiliations:** 1 Department of Neuroscience, Sheffield Institute for Translational Neuroscience, University of Sheffield, Sheffield, United Kingdom; 2 Faculty of Life Sciences, University of Manchester, Manchester, United Kingdom; 3 Medical Research Council Centre for Developmental and Biomedical Genetics, University of Sheffield, Sheffield, United Kingdom; Oregon Health & Science University, United States of America

## Abstract

Charcot-Marie-Tooth disease (CMT) represents a group of neurodegenerative disorders typically characterised by demyelination (CMT1) or distal axon degeneration (CMT2) of motor and sensory neurons. The majority of CMT2 cases are caused by mutations in mitofusin 2 (MFN2); an essential gene encoding a protein responsible for fusion of the mitochondrial outer membrane. The mechanism of action of MFN2 mutations is still not fully resolved. To investigate a role for loss of Mfn2 function in disease we investigated an ENU-induced nonsense mutation in zebrafish MFN2 and characterised the phenotype of these fish at the whole organism, pathological, and subcellular level. We show that unlike mice, loss of MFN2 function in zebrafish leads to an adult onset, progressive phenotype with predominant symptoms of motor dysfunction similar to CMT2. Mutant zebrafish show progressive loss of swimming associated with alterations at the neuro-muscular junction. At the cellular level, we provide direct evidence that mitochondrial transport along axons is perturbed in Mfn2 mutant zebrafish, suggesting that this is a key mechanism of disease in CMT. The progressive phenotype and pathology suggest that zebrafish will be useful for further investigating the disease mechanism and potential treatment of axonal forms of CMT. Our findings support the idea that MFN2 mutation status should be investigated in patients presenting with early-onset recessively inherited axonal CMT.

## Introduction

Mitochondrial morphology depends on regulated fission and fusion events. Mitofusin 2 (MFN2) mediates the fusion of mitochondria, thus it is instrumental in this process [1]. There is emerging evidence that disturbed mitochondrial dynamics contributes to several human diseases, including major neurodegenerative disorders such as Parkinson’s disease [2], [3]. Mutations in MFN2 are known to be the leading cause of axonal Charcot-Marie-Tooth disease (CMT), and are found in patients with CMT type 2A and also in patients with hereditary motor and sensory neuropathy type (HMSN) type VI [4], [5]. The majority of MFN2 mutations are dominantly inherited, however their precise mechanism of action remains controversial.

Gene targeting studies in mice suggest that MFN2 haploinsufficiency is not sufficient to cause a neurological phenotype [6]. On the other hand, gain of function experiments in mice overexpressing a CMT-linked mutant MFN2 allele, demonstrated that this was sufficient to induce a specific neurological phenotype [7]. Despite this, several CMT patients with compound heterozygote MFN2 mutations have been reported, and these all manifested as recessively inherited CMT2A [8]. Thus it is feasible that mutant MFN2 can cause CMT in both a dominant and a recessive fashion, depending on the specific genetic defect. Complete loss of function of MFN2 in gene-targeted mice results in a loss of viability during early embryonic development, thus precluding further investigation of juvenile and adult stages [6]. Recessive MFN2 alleles have been identified as the cause of fetal-onset axonal dystrophy in dogs [9] and fetal-onset neuroaxonal dystrophy in Tyrolean cattle [10]. However in both these cases the mutations were complicated; either a small deletion, or the creation of a cryptic splice site. Therefore we chose to study loss of MFN2 function in a complementary vertebrate model, the zebrafish. Zebrafish are an excellent model organism for studying neurological disorders. They have a well-conserved vertebrate nervous system, and N-ethyl-N-nitrosourea (ENU) induced mutations have so far been generated for approximately 31% of all zebrafish genes (source: Zebrafish Mutation Project http://www.sanger.ac.uk/Projects/D_rerio/zmp/). Furthermore for the study of diseases affecting the nervous system, zebrafish motor and sensory neurons closely resemble those in man, and diseases including spinal muscular atrophy [11], [12], hereditary spastic paraplegia [12], [13] and amyotrophic lateral sclerosis [14], [15] have been successfully modeled using zebrafish.

We describe herein the characterisation of zebrafish bearing an ENU-induced MFN2 nonsense mutation. These zebrafish develop normally but subsequently manifest a progressive motor dysfunction and pathological alterations to the neuro-muscular junction (NMJ), which are associated with defective axonal transport of mitochondria. This vertebrate model supports a role for loss of MFN2 function in the progressive motor dysfunction observed in some CMT type 2A and HMSN type VI patients, and reinforce the importance of evaluating MFN2 mutation state in recessive cases of axonal CMT.

## Materials and Methods

### Zebrafish

All zebrafish were maintained in the University of Sheffield aquaria at 28**°**C. All work with zebrafish was conducted in accordance with UK law, under Project License 40/3323 “Zebrafish models of neurodegenerative disease”, granted to AJG. This process includes review by the local ethical review panel at the University of Sheffield. Mfn2 L285X fish (hu3528) were generated in the Hubrecht Institute (Utrecht, Netherlands), and were obtained from the Zebrafish Mutation Project at the Sanger Centre, Hinxton, Cambridge UK. The L285X mutation generates an additional restriction site for MseI, facilitating genotyping by PCR followed by MseI digest. For survival experiments larvae were raised in tanks containing 50 fish, and a mixture of genotypes. After genotyping by fin-biopsy, fish were housed in tanks containing only a single genotype.

### Behavioral Assays

Swimming endurance was measured in the aquarium using a custom-built swim tunnel apparatus based on one previously used by the authors to characterise mutant SOD1 zebrafish [15]. Critical swimming speed (Ucrit) was calculated as previously described [15], [16], [17]. Swimming posture was recorded by video recording individually housed zebrafish in the aquarium. The recordings were then scored by a blinded investigator, who recorded the duration of any bouts of swimming at more than 30° from horizontal.

In accordance with regulations relating to animal welfare we closely monitored all zebrafish that showed signs of inability to swim. Eventually mutant fish spent most of their time resting on the floor of the tank. The humane endpoint for survival was taken as the point at which these fish lost the ability to remain upright for >50% of the observation time.

### Immunostaining

NMJ staining was conducted as previously described [15]. Images were captured using an SP5 resonant scanning confocal microscope (Leica, UK), and imported into ImageJ [18] for analysis. Intensity correlation quotient (ICQ) [19] and pre- and post-synaptic area were determined using the intensity correlation analysis and particle analysis plugins for ImageJ.

### Primary Neuronal Cultures

At 24 h after fertilisation zebrafish embryos were terminally anaesthetized with tricaine (MS 222) and bleached according to standard procedures [20]. Following bleaching the embryos were dechorinated in sterile 80% Hanks Buffered Saline Solution (Invitrogen), transferred to Ringer’s solution containing 0.25% trypsin, and incubated at 28**°**C for 5 minutes. Embryos were then triturated using fire-polished pasteur pipettes until a suspension of single cells was obtained. Cells were suspended in media containing 80% Lebovitz L15 (Invitrogen), 1× B27 supplement (Invitrogen), 2% horse serum (Sigma), 5 mg/ml Pen/Strep, 10 ng/ml CNTF (R&D systems), 1 ng/ml BDNF (R&D systems), and 100 pg/ml GDNF (R&D systems), and maintained on poly-L-lysine coated 22×22 mm glass coverslips at 28**°**C.

### Live Imaging and Analysis

Zebrafish neurons were imaged and analysed as described previously for mouse neurons [21], [22], except the cultures were maintained at 28**°**C.

### Statistical Analysis

All analysis was performed using GraphPad Prism 6. Details of specific tests are mentioned in the text and figure legends.

## Results

### A Zebrafish MFN2 Loss of Function Mutation

**Figure 1:**
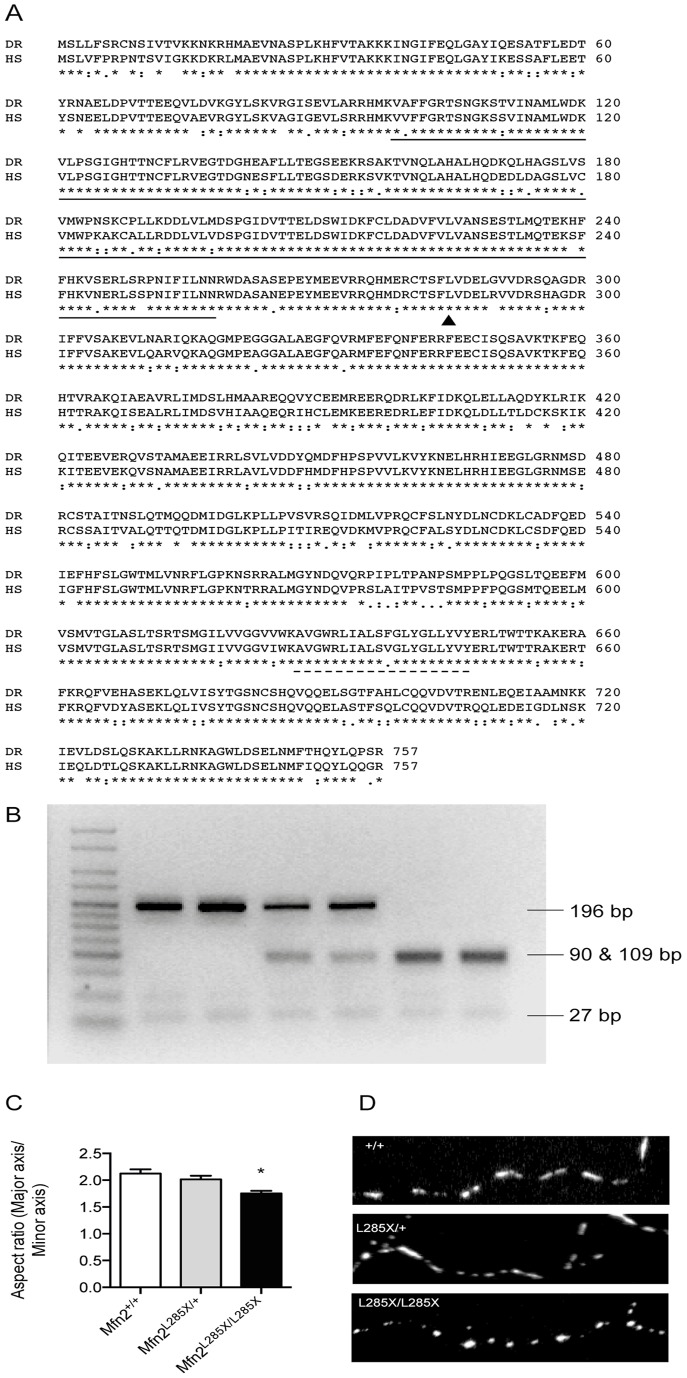
A zebrafish MFN2 mutant. (A) CLUSTAL alignment of zebrafish (Danio rerio: DR) and human (Homo sapiens: HS) Mfn2 protein sequences (Ensembl). The solid and dashed lines indicate the GTPase and transmembrane domains respectively. The site of the ENU induced point mutation (L285) is indicated with an arrowhead. (B) The ENU induced mutation creates a novel restriction enzyme site for MseI, allowing genotyping using PCR followed by MseI digest. The agarose gel shows, from left to right: Hyperladder V (Bioline), two wild type embryos (MFN2^+/+^), two mutation carriers (MFN2^L285X/+^), and two homozygotes (MFN2^L285X/L285X^). (C) Mitochondria in cultured MFN2^L285X/L285X^ neurons have a significantly reduced aspect ratio (p<0.01, Kruskal-Wallis with Dunn’s multiple comparisons test). (D) Representative images of axonal mitochondria stained with Mitotracker Red in cultured neurons of the indicated genotypes.

Human and zebrafish Mfn2 proteins share 82% amino acid identity, and have a highly conserved GTPase domain, transmembrane domain and two heptad coiled coil domains (Figure 1A). A T>A mutation in exon 8 of zebrafish MFN2 was identified in the Zebrafish Mutation Project. This mutation introduces a novel MseI restriction enzyme site allowing genotyping by PCR and MseI enzyme digest (Figure 1B). The mutation leads to the introduction of a stop codon at leucine 285 (MFN2^L285X^), which truncates the protein after the GTPase domain thus removing the transmembrane domain essential for mitochondrial fusion, and therefore produces an Mfn2 protein that is predicted to be non-functional (Figure 1A). Despite the high level of homology, commercial antibodies raised against human Mfn2 failed to recognise the zebrafish protein. Mfn2 plays an important role in mitochondrial fusion events [6]; furthermore we were unable to find evidence for nonsense mediated decay of the mutant mRNA using RT-PCR (data not shown). Therefore in order to seek direct evidence for loss of Mfn2 function we investigated the elongated morphology of mitochondria in the axons of MFN2 mutant zebrafish. To achieve this we derived primary neuronal cultures from 24h MFN2^+/+^, MFN2^L285X/+^, and MFN2^L285X/L285X^ zebrafish embryos, and stained and imaged mitochondria at 7DIV using Mitotracker Red. Morphology was quantified by calculating the aspect ratio, a measure of overall mitochondrial shape calculated by dividing the major axis of a bounding ellipse by the minor axis [23]. The aspect ratio in homozygous mutant neurons was (1.75±0.54), significantly reduced compared to mitochondria in wild type neurons (2.12±0.91) (Figure 1C–D). This result implies that the MFN2^L285X^ mutation behaves as a true loss of function null allele in the zebrafish model, leading to altered mitochondrial dynamics and a change in mitochondrial morphology.

### Characterisation of MFN2 Mutant Zebrafish

**Figure 2:**
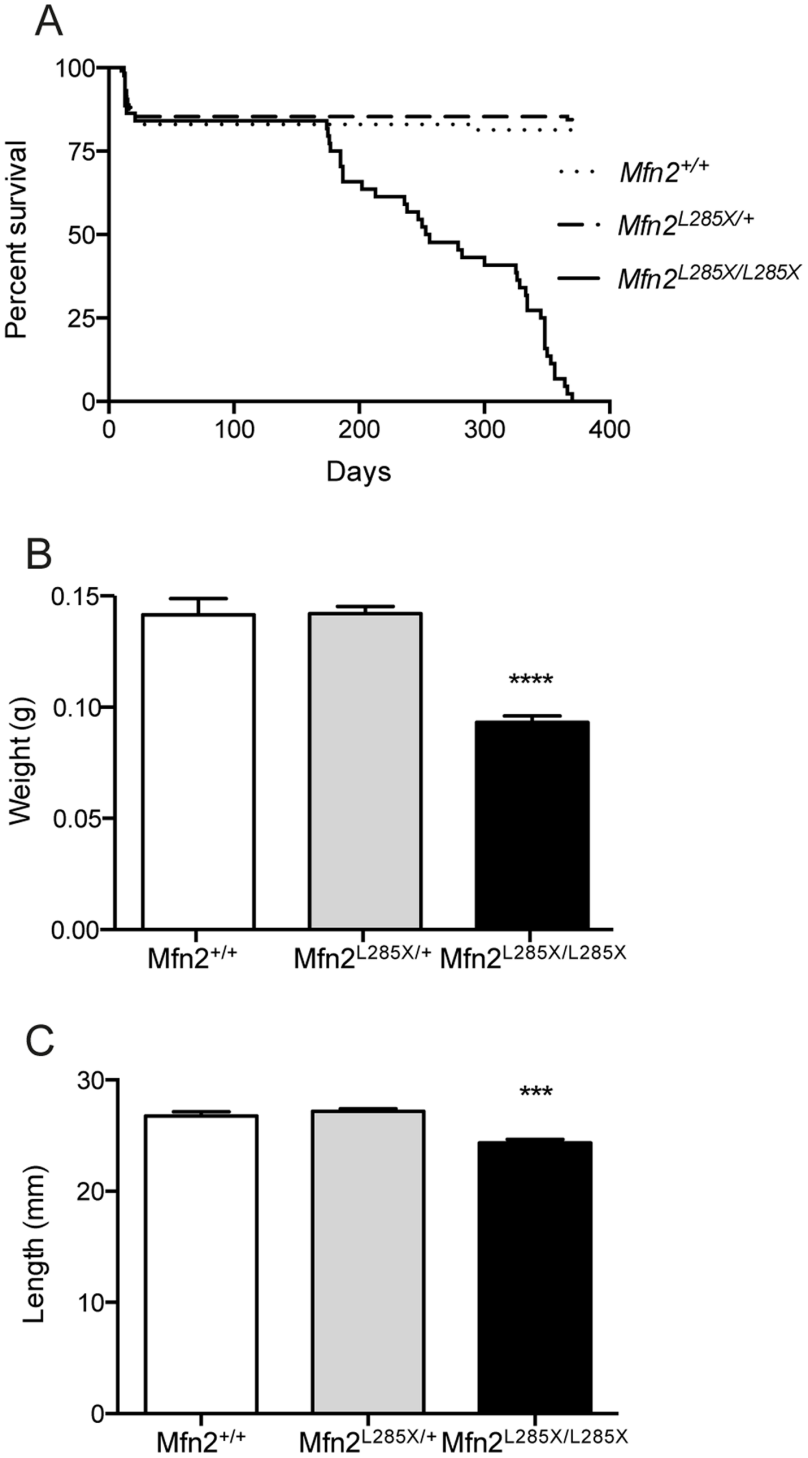
Characterisation of the phenotype of adult MFN2^L285X^ mutant zebrafish. (A) Survival curve showing loss of viability of MFN2^L285X/L285X^ zebrafish from 170 days onwards (p<0.0001, Log-rank (Mantel-Cox) test). (B) Bar graph of body weight for all three groups of siblings at 60–70 days old (p<0.0001, Kruskal-Wallis with Dunn’s multiple comparisons test). (C) Bar graph of the nose to tail length of MFN2^L285X^ mutant and sibling zebrafish at 60–70 days old (p<0.001, Kruskal-Wallis with Dunn’s multiple comparisons test).

MFN2 null mutation leads to early embryonic lethality in mouse due to placental defects [6]. Zebrafish embryos are nourished by a yolk sac and thus have no requirement for a placenta. To explore the possibility that zebrafish MFN2 null mutants will develop normally we studied the progeny of in-crosses between MFN2^L285X/+^ fish. Embryonic MFN2^L285X/L258X^ zebrafish were viable, and both CNS development and swimming behavior was indistinguishable from siblings (data not shown). Since CMT2A patients show disease onset in early or mid-life we carried out an additional MFN2^L285X/+^ in-cross, and closely monitored survival and the phenotype of the offspring (n = 230). There was no difference in survival amongst young adults, all three genotypes showing approximately 85% survival at the time of fin biopsy for genotyping (60–70 days old; Figure 2A). We noticed that MFN2^L285X/L258X^ mutants appeared somewhat smaller their siblings. To test this we recorded the length and weight of all fish prior to fin biopsy. By comparing the length and weight of fish grouped by genotype it was clear that MFN2^L285X/L285X^ fish were significantly smaller that their wild type and heterozygous siblings (Figure 2C–D). We also determined the overall viability of MFN2 mutant zebrafish, and this revealed a gradual decline in survival of MFN2^L285X/L258X^ mutants from 175 days onwards (p<0.0001 Mantel-Cox log-rank test; Figure 2A).

### MFN2 Mutation Leads to Progressive Loss of Motor Function

**Figure 3:**
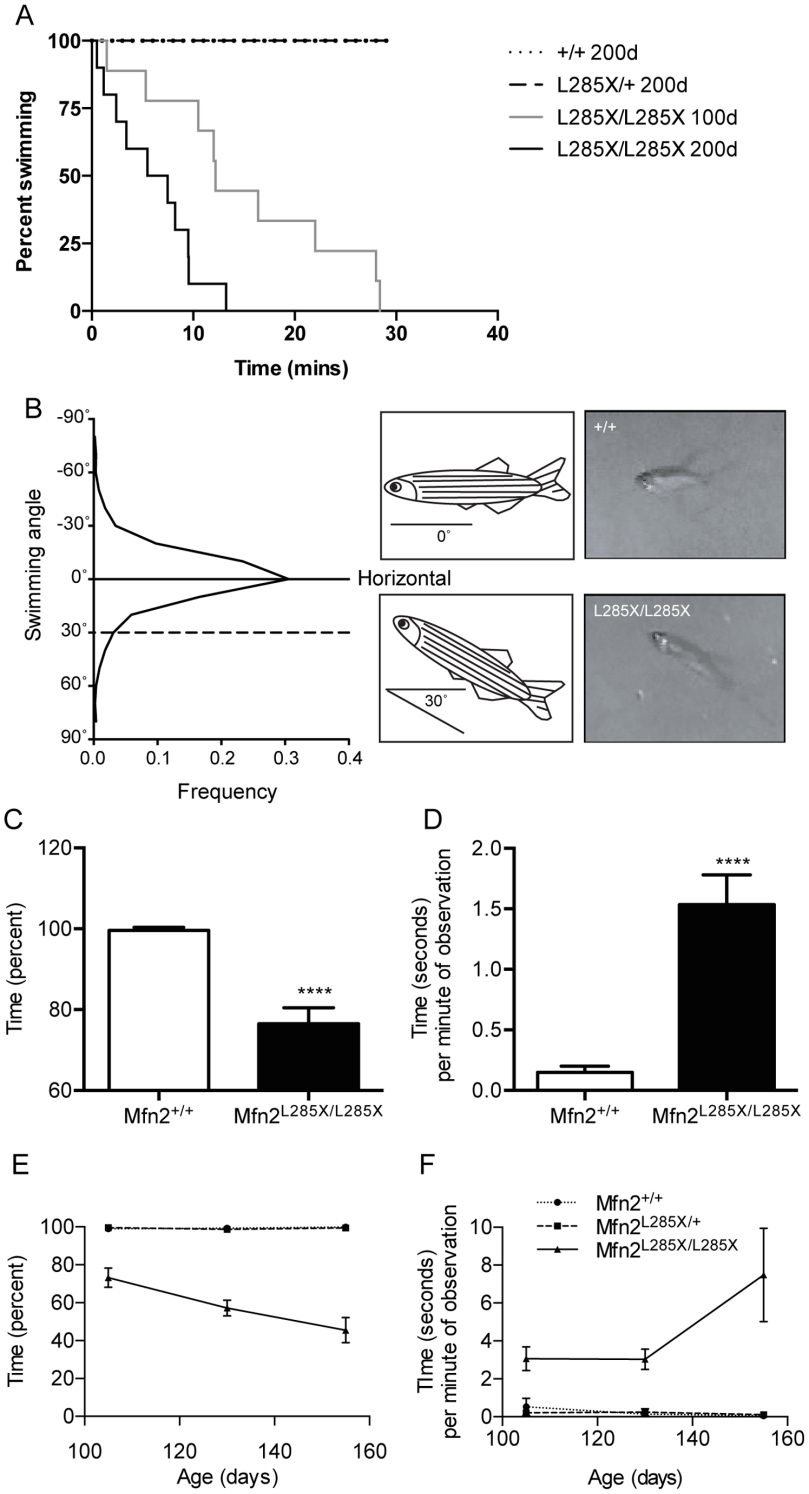
MFN2L285X/L285X zebrafish have a progressive swimming defect. (A) Kaplan-Meier plot of swimming endurance in 100 and 200 day-old zebrafish of each genotype. MFN2+/+ and MFN2^L285X/+^ zebrafish swam for 30 minutes at both 100 and 200d old. For clarity only the 200d data is plotted, although the lines are superimposed. (MFN2^L285X/L285X^: p<0.001 at both ages, Log-rank (Mantel-Cox) test). (B) Frequency distribution plot of swimming angle (measured from horizontal) of adult wild type zebrafish. Representative frames captured from digital video recordings of adult zebrafish and cartoons are shown. The upper panel shows a wild type sibling, and the lower panel shows a MFN2^L285X/L285X^ zebrafish showing abnormal posture, with the body angled at 30**°** from horizontal. (C) Bar graph showing the percentage of time spent with normal swimming posture during 1 minute of observation in 90 day-old zebrafish (p<0.0001, 2-sided T test Mann-Whitney). (D) Bar graph showing the mean duration of each bout of abnormal swimming in 90 day-old old zebrafish (p<0.0001, 2-sided T test Mann-Whitney). (E, F) Graphs showing the decline in normal swimming posture (E) and the increasing duration of each bout of abnormal swimming (F), between 105 and 155 days old.

To determine whether loss of Mfn2 function could lead to motor defects such as those reported in CMT2A we investigated swimming behavior as a read-out of motor function. Close observation of adult MFN2^L285X/L285X^ zebrafish suggested that they displayed altered swimming posture compared to their siblings. Thus we used an established method to determine the critical swimming velocity (Ucrit) of MFN2^+/+^, MFN2^L285X/+^, and MFN2^L285X/L285X^ zebrafish at 100 and 200 days [15], [24]. We deployed a swim tunnel apparatus identical to that described previously [15] to determine the maximum flow rate which fish could swim against for a sustained period. This test is similar in nature to the rotarod test used to measure motor dysfunction in mouse models. The results demonstrate that adult MFN2^L285X/L285X^ zebrafish show a progressive loss of endurance swimming between 100 and 200 days old (Figure 3A). In keeping with this the Ucrit in 100 and 200 day-old MFN2^+/+^ and MFN2^L285X/+^ zebrafish was 28 cm/s, which was significantly reduced to 12.11±7.55 cm/s (mean ± S.D) (p<0.01, Kruskal-Wallis test) in 100 day-old and 4.55±3.23 cm/s (p<0.001) in 200 day-old MFN2^L285X/L285X^ zebrafish.

Freely swimming zebrafish normally maintain a horizontal posture except when swimming to the top or the bottom of the tank. By video recording a side-view of MFN2^+/+^ zebrafish freely swimming in a fish tank we determined the typical swimming angle of adult zebrafish per minute of observation (Figure 3B). The frequency distribution for swimming angle measured between the head and the tail of MFN2^+/+^ fish is Gaussian. MFN2^+/+^ zebrafish spend less than 5% of the time swimming at an angle greater than 30° below horizontal. MFN2^L285X/L258X^ zebrafish were unable to maintain a horizontal posture, and frequently their tail dropped such that they assumed an angled posture (Figure 3B). Post mortem analysis excluded the possibility that this was caused by defective swim bladder inflation. We hypothesised that this altered swimming posture was another outcome of motor impairment in the MFN2^L285X/L258X^ zebrafish, and therefore made video recordings of a cohort of 90 day-old MFN2^+/+^ and MFN2^L285X/L285X^ zebrafish swimming in individual tanks, and then calculated the proportion of time spent swimming normally i.e. at an angle of less than 30° from horizontal (Figure 3C). We also determined the average duration of bouts of abnormal posture, swimming at more than 30° from horizontal (Figure 3D). MFN2^L285X/L285X^ zebrafish exhibited significant alterations in both these parameters, thus we investigated whether the changes in motor performance were progressive by investigating all 3 groups of MFN2^+/+^, MFN2^L285X/+^, and MFN2^L285X/L285X^ zebrafish at 105, 130 and 155 days old (Figures 3E-3F). Only MFN2^L285X/L285X^ zebrafish showed a progressive decline in the percentage of time spent swimming normally (Figure 3E). They also showed an increase in the duration of bouts of abnormal swimming, demonstrating the progressive nature of motor dysfunction in these zebrafish (Figure 3F). Therefore we conclude that loss of Mfn2 function is associated with progressive motor defects in adult zebrafish.

### Loss of Motor Function is Associated Aberrant NMJ Pathology

**Figure 4:**
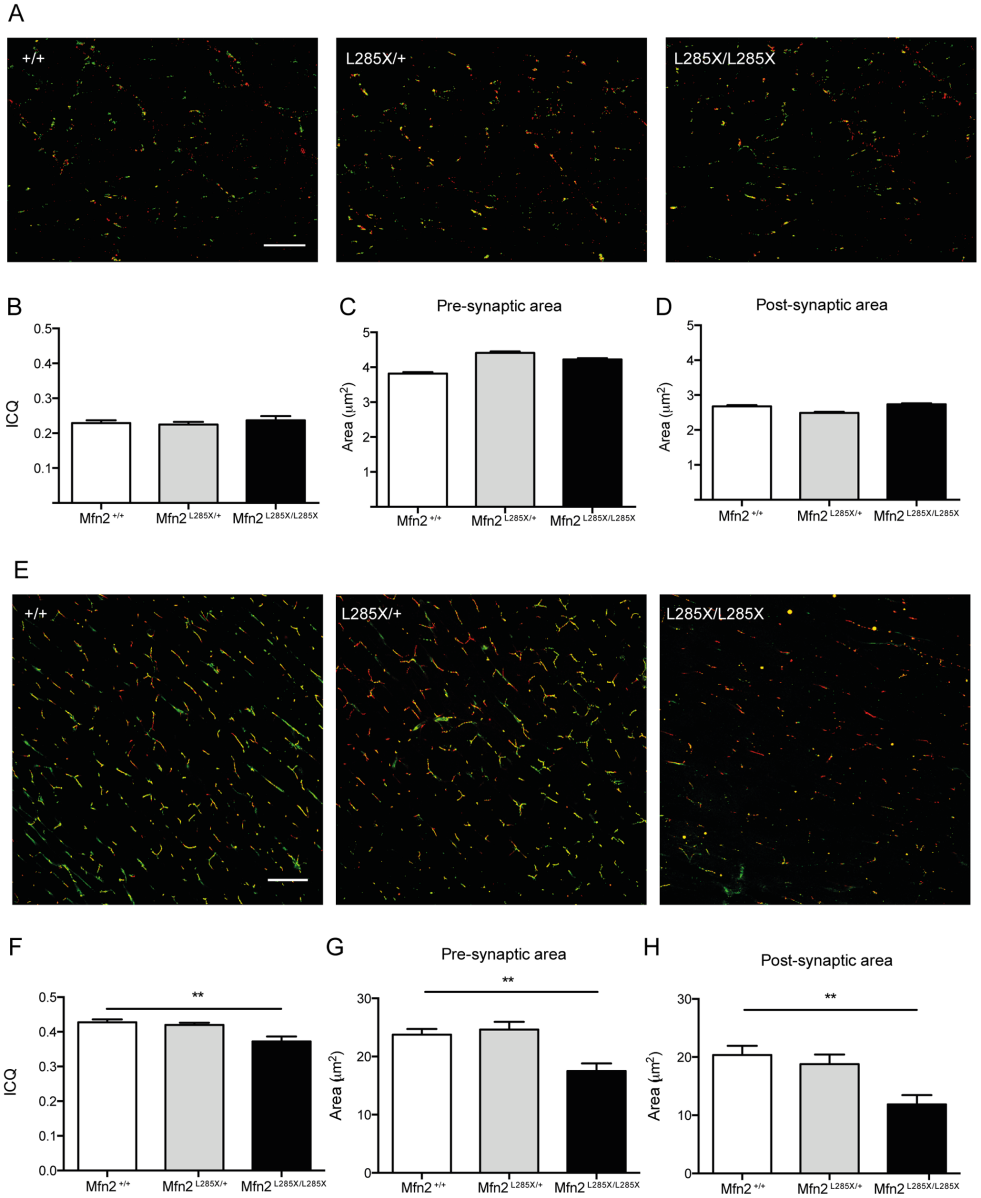
Alterations of NMJ pathology in MFN2^L285X^ larvae (A–D) and adults (E–H). (A) Representative images of dual immunofluorescence staining of whole mount 15d larvae of each genotype for **α**-Bungarotoxin (green) and SV2 (red). (B) ICQ analysis of 3 larvae per group reveals no alteration of SV2/**α**-Bungarotoxin co-localisation in MFN2^L285X/+^ or MFN2^L285X/L285X^ larvae. Compared to siblings the (C) pre-synaptic area, and (D) post-synaptic area are not significantly different in MFN2^L285X/+^ or MFN2^L285X/L285X^ larvae. (E) Representative images of a-Bungarotoxin (green) and SV2 (red) in 200 day-old zebrafish. (F) ICQ is significantly reduced in MFN2^L285X/L285X^ at this stage, and there are significant reductions in the pre- and post-synaptic area (G and H). Scale bars  = 10 **µ**m.

Distal axonopathy is a clinical feature of CMT. To determine whether the altered swimming in MFN2 mutant zebrafish was associated with defects in the distal axon we investigated NMJ pathology in non-symptomatic (15 day-old larvae) and symptomatic (200 day-old adult) zebrafish. Using SV2 as a presynaptic marker that labels motor neurons, and α-bungarotoxin a post-synaptic marker of the NMJ, we used whole mount immunofluorescent staining and confocal microscopy to quantify the intensity correlation quotient (ICQ) [19] and size of pre- and post-synaptic compartments in the trunk musculature. The ICQ is a statistically testable single-value assessment of the relationship between two staining patterns: colocalising signals have 0<ICQ≤+0.5, for random staining ICQ = 0, and for non-colocalising (segregated) staining 0>ICQ≥–0.5 [19]. In 15 day-old mutant zebrafish (Figure 4A) SV2 and **α**-bungarotoxin showed ICQ values of 0.237±0.054, similar to that of controls (Figure 4B), and the pre- and post-synaptic areas (Figure 4C–4D) were also similar in mutants and controls. We found that adult NMJs show increases in both the ICQ and pre/post synaptic area compared to larvae. At 200 days (Figure 4E), when the fish displayed clear alterations in swimming, ICQ was significantly reduced in homozygous mutants compared with controls (0.372±0.043 in mutants and 0.427±0.025 in controls) (Figure 4F) and the area of both pre- and post-synaptic compartments (Figure 4G–4H) was also significantly reduced (p<0.01 one-way ANOVA with Bonferoni’s multiple comparison test). Therefore we conclude that altered swimming in Mfn2 zebrafish is associated with defects at the NMJ.

### Axonal Transport of Mitochondria is Defective in Homozygous MFN2 Mutant Neurons

**Figure 5:**
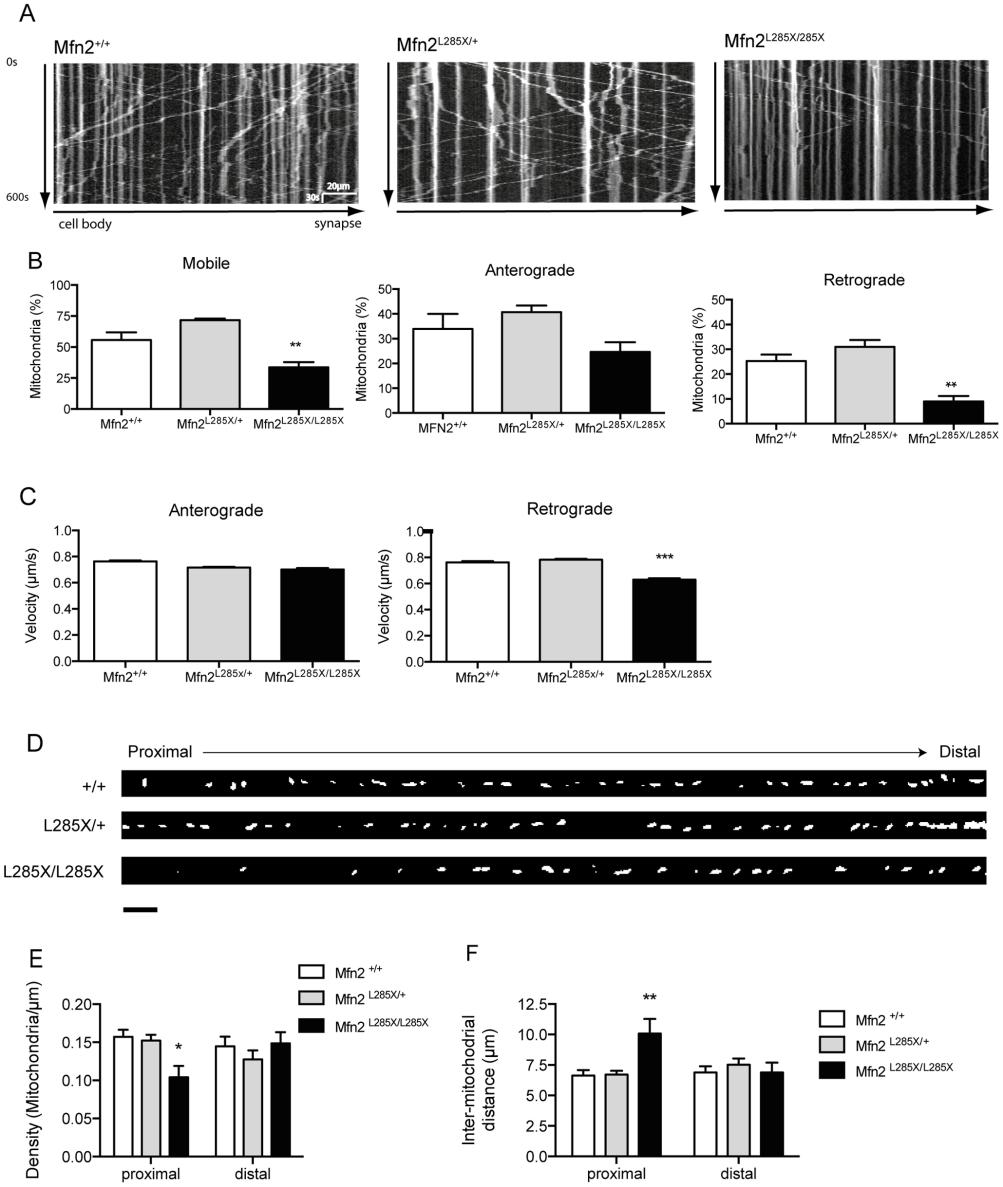
Analysis of axonal transport of mitochondria. (A) Representative kymographs showing defective axonal transport of mitochondria along axons of cultured zebrafish neurons from MFN2^L285X/L285X^ embryos compared with MFN2^+/+^ and MFN2^L285X/+^ neurons. (B) Mitochondria in cultured MFN2^L285X/L285X^ neurons are significantly less motile; anterograde displacement of mitochondria is not significantly altered, but retrograde displacement is significantly reduced (p<0.01, one-way ANOVA). (C) The velocity of retrograde but not anterograde mitochondrial transport is significantly reduced in cultured MFN2^L285X/L285X^ neurons (p<0.001, Kruskal-Wallis with Dunn’s multiple comparisons test). (D) Representative images of axons from neurons of each genotype. The cell body is to the left, and distal axon is to the right hand side (scale bar  = 10 **µ**m) (E) Comparison of mitochondrial density in proximal and distal axonal segments, there is a significant reduction in mitochondrial density in the proximal but not distal segment of MFN2^L285X/L285X^ neurons (p<0.05, 2-way ANOVA with Bonferroni’s multiple comparisons test). (F) There is a significant increase in the inter-mitochondrial distance in the proximal axon of MFN2^L285X/L285X^ neurons (p<0.01, 2-way ANOVA with Bonferroni’s multiple comparisons test).

CMT type 2A affects the distal axons of motor, and to a lesser extent, sensory neurons, which are the longest cells in the human body. We have previously showed that changes in mitochondrial morphology are associated with altered mitochondrial transport [21], [23]. Furthermore loss of MFN2 function in mouse neurons leads to changes in axonal transport of mitochondria [25], [26]. Thus it is possible that MFN2 loss of function in the zebrafish model leads to impaired NMJ pathology and loss of motor function by a mechanism involving disruption of mitochondrial transport along axons. Therefore we used time-lapse imaging of Mitotracker red-labeled mitochondria to quantify mitochondrial transport in cultured neurons obtained from MFN2^L285X^ zebrafish and control siblings. Representative kymographs of mitochondrial transport are shown in Figure 5A. These reveal evidence for a reduction in retrograde transport in the MFN2^L285X/L285X^ neurons. By analyzing time-lapse recordings we confirmed that the number of motile mitochondria is significantly reduced in MFN2^L285X/L285X^ neurons (Figure 5B). This is similar to the reduced mobility of mitochondria reported in dorsal root ganglion neurons derived from MFN2 knockout mice [27]. We investigated the possibility of differential effects on anterograde and retrograde transport of mitochondria, and found that retrograde transport was selectively impaired in MFN2^L285X/L285X^ neurons (P<0.05) (Figure 5B). To understand the basis for this we measured the velocity of mitochondrial transport (Figure 5C), and discovered a significant reduction in the velocity of mitochondria being transported in the retrograde direction. Thus we find evidence for a selective impairment of retrograde mitochondrial transport in the zebrafish model. To determine whether defective axonal transport of mitochondria could be due to degeneration of axons in this model, we investigated the average length of axons in the primary neuron cultures. There were no significant differences in axonal length in any of the cultures (data not shown) suggesting that the mitochondrial transport defects are not arising because of underlying axon degeneration.

### MFN2 Mutation Leads to Altered Mitochondrial Distribution Along Axons

Alterations in mitochondrial transport are predicted to disturb the normal distribution of mitochondria along the axon, as we have previously reported in models of amyotrophic lateral sclerosis and hereditary spastic paraplegia [21], [22]. This defect could underlie axonal dysfunction and the progression of symptoms. In keeping with defective mitochondrial transport in CMT2A, muscle biopsies from patients have revealed an irregular distribution of mitochondria in axons [28]. To investigate whether axonal transport defects cause a similar effect in the zebrafish model we imaged axons from cultured zebrafish neurons, which suggested a change in mitochondrial distribution in the proximal segment of MFN2^L285X/L285X^ neurons (Figure 5D). We therefore analysed the mean mitochondrial density (number of mitochondria per micron of axon), and inter-mitochondrial distance, in proximal and distal axonal segments. This revealed a significant reduction in mitochondrial density and an increase in inter-mitochondrial distance in the proximal, but not the distal, axon (Figure 5D–5E). These results suggest that MFN2^L285X^ specifically affects retrograde transport leading to altered distribution of mitochondria along the axon, consistent with previous observations in CMT2A patient sural nerve biopsies [28].

## Discussion

The zebrafish MFN2 mutant we report here provides important new data relating to the pathogenesis of CMT2A. The majority of MFN2 mutations reported in CMT are inherited in an autosomal dominant fashion [28], however autosomal recessive inheritance has recently been reported in three different families [8]. Autosomal dominant CMT2A has been faithfully modeled by expressing a mutant MFN2 allele in mouse [29]. However to date, MFN2 null models have resulted in early embryonic defects in mice due to aberrant placental development [6], and investigation of MFN2 loss of function in mice has relied on cell specific gene silencing [25], [30]. These studies have provided clear evidence for the importance of MFN2 in Purkinje cells and striatal neurons. If these cell types are affected in CMT patients, then it is at a sub-clinical level. Thus the role of MFN2 null alleles is still not properly understood at the level of the whole organism, which is particularly relevant to better understand the rare recessive MFN2 alleles. We describe a zebrafish MFN2 null allele that causes a progressive loss of motor function that is inherited in an autosomal recessive fashion. In contrast to embryos injected with morpholino oligonucleotides targeting a splice site in MFN2, which show early developmental defects [31], the stable MFN2^L285X^ zebrafish appear healthy as embryos. This is likely to be accounted for by maternal inheritance of MFN2 mRNA and protein in the mutants. We were unable to address this further since homozygous MFN2 mutants were not able to breed successfully. Alternatively Mfn1 function may compensate for Mfn2 defects during embryogenesis. Genomic analysis suggests that zebrafish have two loci encoding orthologues of MFN1. The observation that MFN2^L285X/L285X^ zebrafish are smaller than their siblings is interesting because mutant tardbp zebrafish are also smaller at this stage of development [32]. These findings have implications for husbandry of zebrafish mutants bearing mutations in human neurological disease genes.

Our zebrafish model provides support for loss of MFN2 function in axonal CMT, in particular it is consistent with the idea that the recessive forms of axonal CMT with compound heterozygote MFN2 mutations [8], are caused by substantially reduced, or complete loss of Mfn2 function. Because of the existence of dominant and recessively inherited mutations in MFN2, it now seems possible that MFN2 leads to CMT through both gain and loss of function mechanisms. This idea is supported by evidence for disrupted mitochondrial transport in the MFN2^L285X/L285X^ zebrafish, which was also reported in embryonic neurons cultured from the mouse MFN2 knock-out, and the mouse transgenic gain of function model [7], [25].

Since the principal target of the L285X mutation is mitochondria we investigated mitochondrial transport in this model. Quantitative analysis of mitochondrial motility revealed significant reductions in both the frequency and velocity of retrograde mitochondrial movements. It has previously been shown in mouse that Mfn2 interacts with Miro and that silencing of Miro replicates the effect of Mfn2 knockout on axonal transport [27]. Miro is known to interact with anterograde (kinesin) motors, however the relationship between anterograde and retrograde motors and the net rate of transport is complex, thus it is possible that disruption of Mfn2/Miro in the L285X zebrafish model contributes to the observed retrograde transport defects.

The MFN2^L285X^ model offers an opportunity for further research into recessive CMT associated with compound heterozygote MFN2 mutation. Zebrafish models are proving to be useful for identifying novel therapeutic approaches, either by directly testing candidate drugs [33], or by leveraging the power of genetic screens to identify novel disease modifier genes [34], which will lead to new avenues of therapy development.
